# The Complete Plastome Sequence Of *Penstemon fruticosus* (Pursh) Greene (Plantaginaceae)

**DOI:** 10.1080/23802359.2017.1398620

**Published:** 2017-11-06

**Authors:** Nathan J. Ricks, Jason M. Stettler, Mikel R. Stevens

**Affiliations:** Department of Plant and Wildlife Science, Brigham Young University, Provo, UT, USA

**Keywords:** *Penstemon fruticosus*, chloroplast genome, phylogenetic analysis

## Abstract

The genus *Penstemon* is an emerging model for the study of continental adaptive radiation. We report here the first complete plastome sequence for this genus. The *P. fruticosus* (shrubby, or brush penstemon) plastome is 152,704 bp in length with a quadripartite structure consisting of a large single-copy region (83,693 bp) and a small single-copy region (17,820 bp) that are separated by two inverted repeats (25,594 bp). The plastome contained 24 tRNA genes, 8 rRNA genes, and 83 protein-coding genes for a total of 115 unique genes. Phylogenetic analysis of whole chloroplast sequences shows that the nearest relatives of *P. fruticosus* are the *Plantago* and *Veronica* genera in the Plantaginaceae family.

*Penstemon* (Mitchell) is native to North America with a distribution that reaches from the Arctic Circle to the tropics of Central America. This genus is an emerging model for continental adaptive radiation with over 270 species that are adapted for specialized ecological niches of North America (Straw 1966; Wolfe et al. 2006). The rapid speciation and diversification of *Penstemon* has created a challenge for accurate phylogenetic analysis using conventional molecular markers (Wolfe et al. 2006; Blischak et al. 2014; Wessinger et al. 2016). Bush, or shrubby penstemon (*P. fruticosus* (Pursh) Greene [Plantaginaceae]), is native to the United States Intermountain West, and is found within *Penstemon*’*s* most basal clade (Datwyler and Wolfe 2004). Our data will be the first complete plastome for this genus, and will aid in future evolutionary and systematic studies of this genus.

*Penstemon fruticosus* plants originating from vegetative cuttings were collected near Bogus Basin Resort, ID [43°47′45.060″N, –116°05′49.956″W] for this study. Voucher specimens (BRY197292) have been deposited in the Stanley L. Welch Herbarium (BRY), Brigham Young University, Provo, UT, USA. DNA was extracted using a modified CTAB method (Doyle 1987). We generated the plastome sequences using the paired-end (2 × 250 bp) Illumina HiSeq platform (Illumina Inc., San Diego, CA). The programs PEAR (Zhang et al. 2013), NOVOPlasty (Dierckxsens et al. 2017), CpGAVAS (Liu et al. 2012), MISA (Thiel 2003), and REPuter (Kurtz and Schleiermacher 1999) were used to align paired end reads, assemble, annotate, identify SSR’s, and identify repetitive sequences, respectfully. A maximum likelihood (ML) phylogenetic analysis was performed using Mega7 (Kumar et al. 2016) using plastomes of 13 species obtained from NCBI.

The plastome of bush penstemon was 152,704 bp in length with a quadripartite structure, consisting of a large single-copy region (83,693 bp) and a small single-copy region (17,820 bp) that are separated by two inverted repeats (25,594 bp), and an average CG content of 37.9% (GenBank accession number MG201976). The average sequence coverage was 9449*x*. The plastome contains 115 unique genes (24 tRNA genes, 8 rRNA genes, and 83 protein-coding genes). We identified 20 simple sequence repeat (SSR) loci of which 9, 2, and 9 were mono-SSR, tri-SSR, and tetra-SSR loci, respectively. A total of 22 forward and 32 palindrome repeats were identified; however, no reverse complement repeats were identified.

To determine the phylogenetic position of *P. fruticosus* within the order Lamailes, we constructed a ML phylogeny with 1000 bootstrap replicates using plastome alignments of 12 Lamailes species, with *Solanum lycopersicum* as an outgroup (Figure 1). Our phylogenetic analysis is consistent with phylogenies of Lamailes that used single plastid genes (Albach et al. 2005) and multiple concatenated genes (Yi and Kim 2016). The *Penstemon* genus is an outgroup to the sister clades formed by the *Plantago, Veronica,* and *Veronicastrum* genera of the Plantaginaceae family. The complete plastome of *P. fruticosus* provides genetic data critical for the study of evolution and systematics of *Penstemon* as a model for continental adaptive radiation.

**Figure 1.**
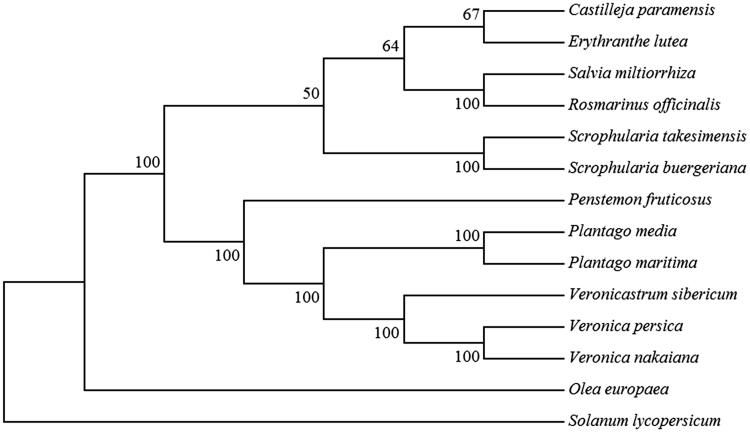
Maximum-likelihood phylogenetic tree of *P. fruticosus* with 12 species from the order Lamailes with *Solanum lycopersicum* as outgroup. The node numbering represents the bootstrap values from 1000 replicates. Accession numbers are listed as below: *Castilleja paramensis* NC_031805.1, *Erythranthe lutea* NC_030212.1, *Salvia miltiorrhiza* NC_020431.1, *Rosmarinus officinalis* NC_027259.1, *Scrophularia takesimensis* NC_026202.1, *S. buergeriana* NC_031437.1, *Plantago media*NC_028520.1, *P. maritima* NC_028519.1, *Veronicastrum sibiricum* NC_031345.1, *Veronica persica* NC_031344.1, *V. nakaiana* NC_031153.1, *Olea europaea* NC_013707.2, and *Solanum lycopersicum* NC_007898.3.
